# A novel hypothesis-unbiased method for Gene Ontology enrichment based on transcriptome data

**DOI:** 10.1371/journal.pone.0170486

**Published:** 2017-02-15

**Authors:** Mario Fruzangohar, Esmaeil Ebrahimie, David L. Adelson

**Affiliations:** 1 School of Biological Sciences, The University of Adelaide, Adelaide, South Australia, Australia; 2 School of Agriculture, Food and Wine, The University of Adelaide, Adelaide, South Australia, Australia; 3 Australian Centre for Antimicrobial Resistance Ecology, School of Animal and Veterinary Sciences, The University of Adelaide, Adelaide, South Australia, Australia; 4 School of Medicine, Faculty of Health Sciences, The University of Adelaide, Adelaide, Australia; 5 School of Information Technology and Mathematical Sciences, Division of Information Technology, Engineering and the Environment, University of South Australia, Adelaide, Australia; 6 School of Biological Sciences, Faculty of Science and Engineering, Flinders University, Adelaide, Australia; 7 Zhendong Australia – China Centre for Molecular Chinese Medicine, The University of Adelaide, Adelaide, South Australia, Australia; Mayo Clinic Arizona, UNITED STATES

## Abstract

Gene Ontology (GO) classification of statistically significantly differentially expressed genes is commonly used to interpret transcriptomics data as a part of functional genomic analysis. In this approach, all significantly expressed genes contribute equally to the final GO classification regardless of their actual expression levels. Gene expression levels can significantly affect protein production and hence should be reflected in GO term enrichment. Genes with low expression levels can also participate in GO term enrichment through cumulative effects. In this report, we have introduced a new GO enrichment method that is suitable for multiple samples and time series experiments that uses a statistical outlier test to detect GO categories with special patterns of variation that can potentially identify candidate biological mechanisms. To demonstrate the value of our approach, we have performed two case studies. Whole transcriptome expression profiles of *Salmonella enteritidis* and Alzheimer’s disease (AD) were analysed in order to determine GO term enrichment across the entire transcriptome instead of a subset of differentially expressed genes used in traditional GO analysis. Our result highlights the key role of inflammation related functional groups in AD pathology as granulocyte colony-stimulating factor receptor binding, neuromedin U binding, and interleukin were remarkably upregulated in AD brain when all using all of the gene expression data in the transcriptome. Mitochondrial components and the molybdopterin synthase complex were identified as potential key cellular components involved in AD pathology.

## Introduction

Classifying genes into distinct functional groups through Gene Ontology (GO) is a commonly used and powerful tool for understanding functional genomics and the underlying molecular pathways. The functional genomic changes in bacterial pathogens during disease progression or in emerging highly pathogenic strains are poorly understood.

GO analysis commonly begins with enrichment carried out on a short list of genes with statistically significant differential expression [1–3]. In this method, GO term frequencies in the differentially expressed list of genes are compared to a background control, either GO term frequencies of the whole genome, or another list of genes.

This comparison is usually performed using a one sided Fisher-Exact test or a Hypergeometric distribution. This method is called over-representation analysis (ORA) and is implemented nearly in all current GO analysis tools [3–6].

Using routine GO analysis considers all selected genes contribute equally in the final GO classification. The major limitation to the approach is that the original levels of gene expression can significantly affect protein production and consequently actual GO term enrichment. In addition, genes with low or non-differentially expressed values can participate in final GO enrichment through cumulative effects.

The second limitation of traditional ORA analysis is that it can compare just two samples at a time, but in many situations we need to compare GO enrichments of more than two samples. For example, comparing multiple treatment samples to a control sample, comparing time series of samples from the same tissue and the same species. All of these multi-sample comparisons can help us to better understand causative and conserved biological pathways.

To be able to compare GO enrichments of multiple samples we need a robust statistical framework. To our knowledge, Gene Set Enrichment Analysis (GSEA) [7] is the only well described enrichment method that can be applied to multiple samples. This method and its derivatives has been implemented and tested extensively [7–9]. In this method, expression dataset D with N genes and K samples and their phenotype values/classes are given as input data. Given a set of genes S defined as prior biological knowledge, this method can detect if gene set S is significantly enriched by the input data. In this method, first correlation between each gene and phenotype classes is estimated, then genes are sorted based on the absolute values of the correlations. A cumulative statistic similar to Kolmogorov-Smirnov is calculated for gene set S as the enrichment score. If the enrichment score is higher than a given threshold, gene set S can then be considered as belonging to a significant pathway. The method can be useful for some applications, but it has some limitations in comparative analysis.

The first limitation is that the method depends on a measurable phenotypic value for each sample in order to better estimate correlations and sort the genes. In many applications, the expression profiles of multiple samples have no measurable phenotype, similar to the case studies we described in this study.

Furthermore, GSEA merges expression profiles of K samples into one single sorted gene list. Merging samples eliminate the ability to account for the dynamics and variation pattern of expression profiles across samples. As a result, changing the order of the samples produces the same final sorted gene list. But in many biological studies, especially in time series studies, changing the order of samples can result in different biological interpretations, hence merging is problematic. Availability of Gene Set databases provided by Molecular Signature Database (MSigDB) for all species is another limitation for GSEA analysis. To date, MSigDB only contains gene sets for *Danio rerio*, *Homo sapiens*, *Macac mulatta*, *Mus muculus* and *Rattus norvegicus*.

In order to overcome the limitations of GSEA, we developed an approach to estimate and visualise multiple samples’ GO enrichments using their mRNA levels. Our method uses a metric that can identify the most significant biological process(es) or molecular function(s) in a multi sample experiment. We have also developed flexible reports to visualise variation of GO terms across multiple samples.

In this study we show for the first time how mRNA expression levels in bacteria and human can be used to better estimate GO term enrichments. By using mRNA expression levels as coefficients, we are able to consider the impact of low expression level and non-differentially expressed genes such as transcription factors in GO enrichment which are normally discarded in analysis. Furthermore, our approach provides the opportunity to enrich GO terms from the entire transcriptome genome (instead of samples of a short list of genes) and enables us to compare GO enrichments of entire transcriptomes across multiple biological samples.

We implemented the new enrichment method and visual reports on a web server accessible at http://www.comparativego.com. We have used the latest web and database technology (PHP and PostgreSQL) to implement the methods. We are committed to updating the web server database every 12 months. The web server has been tested extensively by different groups from University of Adelaide and worldwide. We recently added support for GO information related to selected eukaryotes including human, zebra fish and yeast.

Bacteria are attractive organisms for GO analysis since they have less post-transcriptional gene silencing compared to animals and plants [10] with mRNA expression levels moderately correlated with protein levels [11]. As the first case study, we applied the new enrichment method to whole transcriptome expression profiling to compare low and high pathogenic strains of one important bacterial pathogen, *Salmonella enteritidis* [12]. The analysis revealed a high level of bacterial-type flagellum-dependent cell motility in the highly pathogenic strain. This mechanism has been well described in *E*. *coli*, but was not reported in the original work on *S*. *enteritidis* [12].

As a eukaryotic case study, we employed whole transcriptome GO analysis to profile Alzheimer’s Disease (AD) pathology. AD, as the leading cause of dementia, is a major concern worldwide with more than 35 million people affected [13]. There is still no effective treatment available and all therapeutic drugs have failed to show efficacy at the clinical level for individuals with AD symptoms [13]. Whole transcriptome GO analysis that takes into account gene expression levels helped us to develop a novel hypothesis for the molecular mechanisms of AD. We also performed GSEA analysis on this case study dataset and compared its result to our method.

## Materials and methods

### Incorporation of mRNA expression levels into GO enrichment

Given N *genes (g*_*1*_…*g*_*n*_*)* in K samples, we estimate the enrichment score *(ES)* of a GO term *t* in sample *s ES*_*t*,*s*_, when expression levels are given as RPKM (Reads per Kilo base per Million Reads)/FPKM (Fragments per Kilo base per Million Reads):

$$ES_{t,s}=\sum_{i=1}^{n}{\text{log}}_{2}[e(i,s)+1]\times I(i,t)$$ (1.1)

or as microarray log fold change:

$$ES_{t,s}=\sum_{i=1}^{n}{\text{log}}_{2}(2^{e(i,s)}+1)$$ (1.2)

Where *e(i*,*s)* is the expression level of *gene g*_*i*_ in sample *s* and *I(i*,*t)* is:

$$I(i,t)={\{}_{0,\;otherwise}^{1,\;if\;g_{i}\;annotated\;by\;GO\;term\;(t)}$$

We then define an intermediate value for fold change (*F*) of GO term *t* from sample *s* to sample *s*+1 (*F*_*t*,*s*_):

ESt,s+1ESt,s (2)

Finally, the average fold change of GO term *t* across all samples is defined as:

$$F_{t}=\sqrt[{k-1}]{\prod_{s=1}^{k-1}F_{t,s}}$$ (3.1)

or log transformed as:

$$F_{t}=\frac{1}{k-1}\sum_{s=1}^{k-1}{\text{log}}_{2}F_{t,s}$$ (3.2)

In general, the most significant GO term associated with an observed expression profile is the one with significantly higher/lower average fold change. It can be identified by an outlier test such as the Grubbs outlier test [14].

GO enrichment is initially estimated at the last (most detailed) level of the GO tree. If there is no significant GO term detected at this level, higher levels (more general levels) of the GO tree are recursively searched until either a significantly represented GO term is found or the highest level of the tree is reached.

Average fold change *F*_*t*_ is sensitive to the order of samples. For example, if we reorder two samples different intermediate fold change values will occur (eq 2). Consequently, the average fold change *F*_*t*_ will change (eq 3).

We also report specific patterns such as GO terms with consistently increasing or decreasing enrichment score between every two consecutive samples:

$$\forall s\in \left[1..k\right),\;ES_{t,s}\leq ES_{t,s+1}$$

### GO enrichment proportions versus GO enrichment scores

In sample *s*, the ratio of the enrichment score of GO term *t* to the total of enrichment scores of all GO terms (*t*_*1*_….*t*_*m*_) can be considered as the GO enrichment proportion (*EP*) of the GO term:

$$EP_{t,s}=\frac{ES_{t,s}}{\sum_{i=1}^{m}ES_{t_{i},s}}\;\Rightarrow \;\sum_{i=1}^{m}EP_{t_{i},s}=1$$

GO enrichment proportions are displayed as pie charts on our webserver.

### Hypothesis testing tool

Although average fold change and other patterns described in the previous section can detect some patterns in individual GO terms, they cannot tell us whether overall GO term enrichment has significantly changed between two samples. We therefore implemented an integrated tool on the web server to test the hypothesis of a significant difference between 2 genome/sample GO term enrichment distributions. Specifically, we implemented a Chi-Square test for 2 samples in R [15] and we compared it with the Kolmogorov–Smirnov test [16] for 2 samples. Both tests are non-parametric and are suitable for comparing 2 lists of paired numbers like GO term enrichment scores/proportions between 2 samples.

In order to use these tests, samples were binned based on GO terms (one GO term was treated as one bin), and for each bin, the enrichment score of related GO terms were considered as the count for that bin.

### Web application

Methods and algorithms were implemented in our web application [17] using PHP 5 and a PostgreSQL database, running on an Apache webserver in a Linux Fedora environment.

### Case study data sets

To demonstrate the biological application of these new methods in global transcriptome GO analysis, expression profiles from two published experiments were used.

The first case study [12] was RNA-Seq global transcriptome data from six strains of *Salmonella enteritidis*, where 3 highly pathogenic strains and 3 low pathogenic strains were compared. The average whole genome expression (RPKM) of 4402 genes of the 3 low pathogenic strains and 3 highly pathogenic strains are presented in S1 File.

For the second case study, whole transcriptome (RNA-Seq) data of AD and normal brains were obtained from Twine et al., 2011 [18]. The RNA was obtained from post-mortem total brains of human normal and AD brains (ID at DNA Data Bank of Japan: SRP004879). The RNA-Seq data was analysed using CLC Genomics workbench (QIAGEN, Finland). Mapping was performed using the following parameter values: mismatch cost: 2, insertion cost: 3, deletion cost: 3, length fraction: 0.8, and similarity fraction: 0.8. RPKM, as expression value, was calculated for 57,773 genes based on the *Homo sapiens* (hg19) reference genome in AD and normal brains. In AD brain samples, 14,720,798 short reads were analysed; 99.98% of the reads were mapped to the reference genome (68.88% to exons and 31.12% to introns). In normal samples, 13,440,858 reads were analysed; 100% of reads were mapped to reference genome (79.27% to exons and 20.73% to introns). Results are presented in S2 File.

To compare the result of our method to GSEA, we used Broad Institute Java application (http://www.broadinstitute.org/gsea). Different parameter sets were tried and following parameters achieved the best result: Permutation Type: Gene Set; Enrichment Statistics; Classic, Metric for Ranking Genes; Ratio of Classes.

## Results

### Introduction of mRNA expression levels into GO analysis

Combining expression profile data with GO term enrichment provided the opportunity to (a) quantify more accurate GO enrichments, (b) extend analysis coverage from sample-wide to genome-wide, and (c) compare GO enrichments of the same list of genes in multiple biological conditions. By considering the influences of all expressed genes in functional genomics, even those with low levels of expression, we increased the accuracy of GO term analysis.

We have also demonstrated that through the use of an outlier test, we can detect GO terms with extreme variation patterns between samples, indicating possible association with the underlying pathway.

### Web application enhancement

Because of the additional computational expense associated with the analysis of the GO distribution of all expressed genes within a genome (global transcriptomics), significant memory and processing resources were required by the Apache web server. To enhance performance and husband system resources we implemented file based caching technology to cache the whole genome GO graphs. When a GO graph is built for the first time, subsequent references to that GO graph, even by other users, are instantaneous. For a better user experience in web applications where long running tasks were performed, we used Ajax technology to implement real time progress bars.

### Case studies

#### Case study 1: Comparison of whole transcriptome based GO enrichment between minimally and highly pathogenic *Salmonella enteritidis*

We used RNA-Seq data for six *Salmonella enteritidis* [12] strains. For each gene in both groups of strains the RPKM counts were averaged.

**Fig 1:**
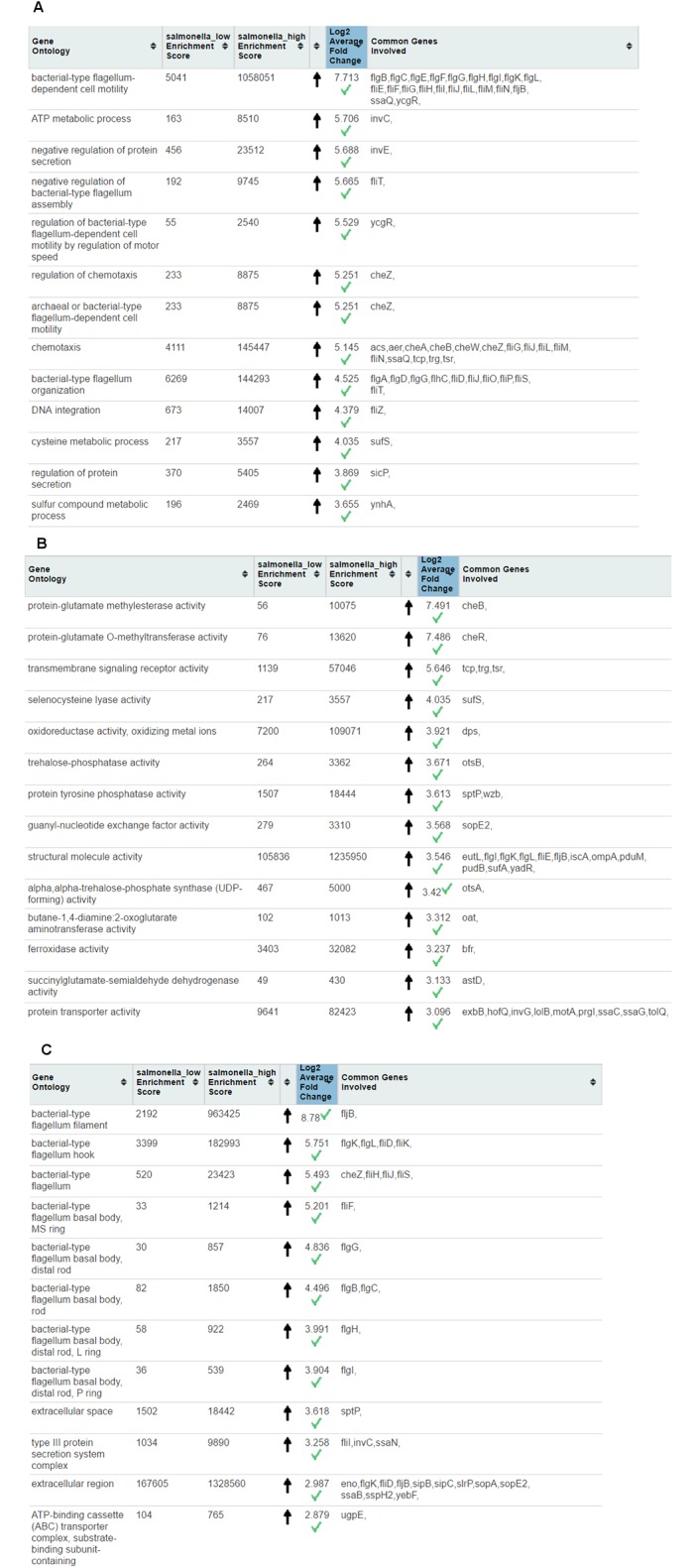
Webserver screenshot from outlier test performed on whole transcriptome of low and high pathogenicity of *Salmonella enteritidis*. (A) Biological Process (B) Molecular Function (C) Cellular Component.

After submission of both gene lists to the web server, whole transcriptome GO enrichment analysis followed by outlier test was performed (enrichment values and outlier test result is shown in Fig 1). In both Biological Process (BP) and Cellular Component (CC), GOs related to bacterial-type flagellum-dependent cell motility (governed by genes such as *flgB* and *flgC*) were the major differentiating functions between high and low pathogenicity *S*. *enteritidis*. Flagellated bacteria such as Salmonella and *E*. *coli* are more mobile and can swim faster. The reversible rotary motor, powered by an ion flux [19], is a significant advantage for bacteria as it provides a tool to rapidly respond to environmental signals and escaping harsh conditions and antibiotics. Interestingly, the GO of “regulation of bacterial-type flagellum-dependent cell motility by regulation of motor speed” (GO ID: 71945, governed by *ycgR* gene) was up-regulated 5.5 fold.

Chemotaxis is another biological process associated with highly pathogenic *S*. *enteritidis* that was upregulated by more than 5 fold. Genes such as *cheA*, *cheB*, *cheW*, and *cheZ* are central in chemotaxis.

In terms of molecular function, highly pathogenic *S*. *enteritidis* increase protein-glutamate methylesterase by 8 fold. Protein-glutamate methylesterase is a molecular function in a two-component regulatory system and is regulated by *CheA* and *CheB*. It has been reported that upregulation of protein-glutamate methylesterase and *CheB* significantly contribute in increasing swimming motility and flagella synthesis in *E*. *coli* [20]. Genetic elements involved in motility are associated with pathogenicity [21]. In addition, it has been demonstrated that these mobile genetic elements (transposons, integrons) increase virulence in animal models as well as colonisation success [21].

#### Case study 2: Comparison of whole transcriptome based GO enrichment between AD and normal human brain

**Fig 2:**
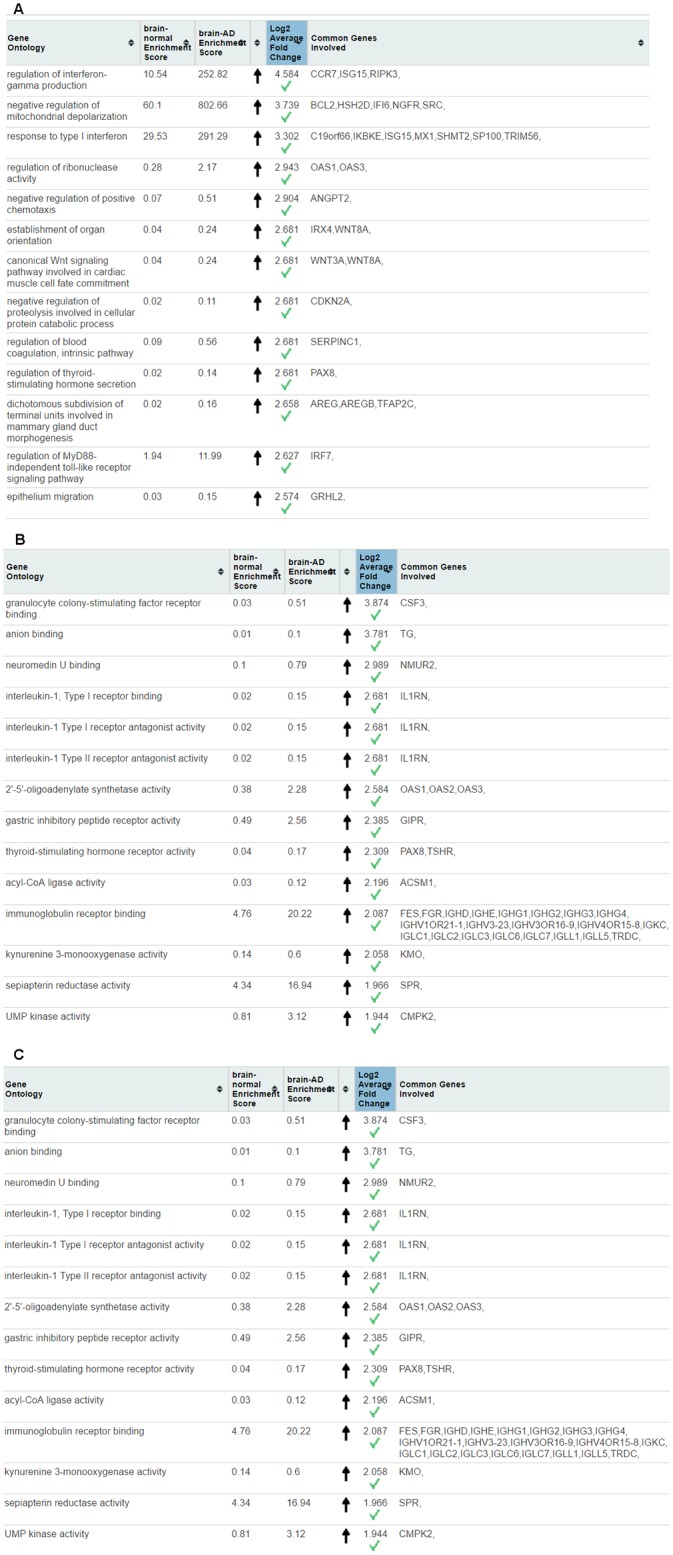
Webserver screenshot from outlier test performed on whole transcriptome of normal and Alzheimer’s disease of *human* samples. (A) Biological Process (B) Molecular Function (C) Cellular Component.

The results of whole transcriptome GO classification followed by outlier testing in AD and normal brains in Biological Process, Molecular Function and Cellular Component are presented in Fig 2. The most significant functions in AD were inflammation and fatty acid related functions including granulocyte colony-stimulating factor receptor binding, interleukin-1, alcohol dehydrogenase, neuromedin U receptor activity, and norepinephrine transmembrane transporter activity.

Log_2_ fold change upregulation of granulocyte colony-stimulating factor receptor binding of AD compared to normal condition is 3.59 (in Molecular Function term). *CSF3* (Colony-stimulating factor-3) is the key member of this functional group. Interleukin-1, Type I receptor binding was another inflammation upregulated function which is highly enriched in AD brain (log2 fold change = 2.59, central gene = *IL1RN*).

Cellular components such as the IgA immunoglobulin complex (*IGHA1*, *IGHA2*, and *IGJ*) endoplasmic reticulum membrane (*DHRS7C*, *RHO*), vesicle lumen (*APOB*), and mitochondrial segments (mainly governed by *Bcl3*) were clearly upregulated in AD which highlight the involvement of mitochondria, endoplasmic reticulum, and vesicle formation in AD. Complexes such as Bcl3/NF-kappaB2 complex (governed by *BCL3* and *NFKB2*), vacuolar lumen (governed by *CLN5*), IPAF inflammasome complex (CASP1, CASP4, NLRC4) help to understand the involvement of inflammation and vascular disorder in AD.

**Fig 3:**
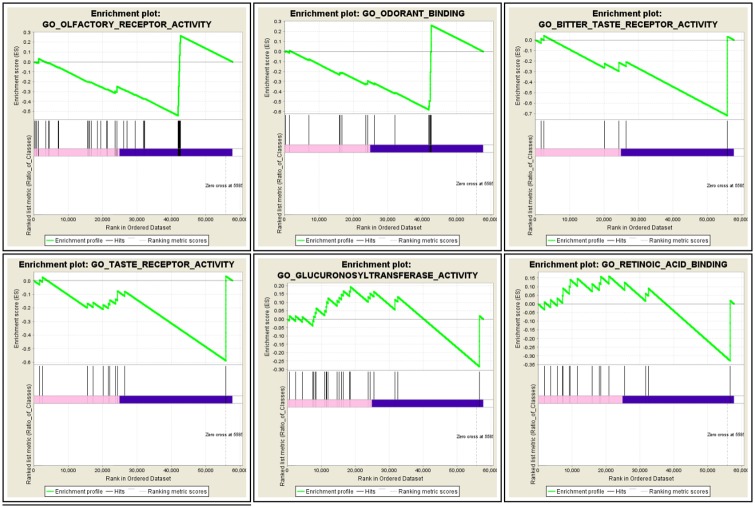
Snapshot of GSEA enrichment result related to molecular function detected in AD.

**Fig 4:**
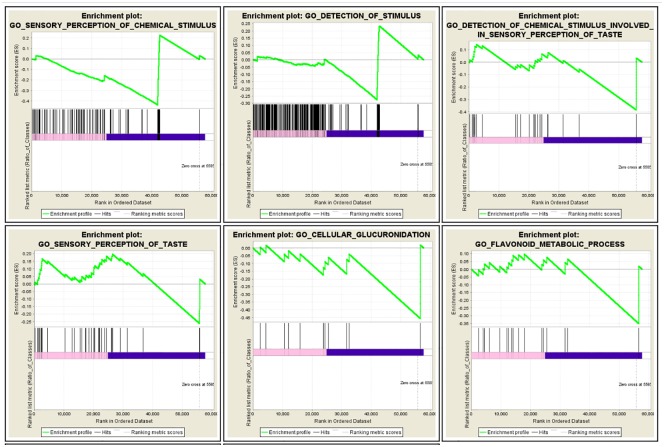
Snapshot of GSEA enrichment result related to biological process detected in AD.

GSEA analysis was performed for whole genome of normal and AD samples using tools and parameter set explained in Material and Method. Significant Molecular Functions and Biological Process in AD phenotype are shown in Figs 3 and 4 respectively.

Full result of GSEA is available in S3 File.

Interestingly, molecular functions related to olfactory and sensory receptors stood out in GSEA analysis. There are numerous publications that identified olfactory deficit as early marker of AD at clinical level [22, 23]. At pathological level, several studies [24, 25] have also shown abnormal cellular pattern of the entorhinal cortex and olfactory neurons that disrupt memory function.

## Discussion

In this study we showed for the first time how mRNA expression levels in bacteria and human could be used to estimate GO term enrichments. By using mRNA expression levels as coefficients, we were able to include the impact of non-significantly expressed genes in GO enrichment. Furthermore, our approach provided the opportunity to enrich GO terms at the entire transcriptome level (rather than a subset of genes) across multiple biological conditions. The outlier test also detected significant patterns in the data.

Unlike a previous GSEA method [7], our method is independent of phenotypic data and also reflects the order of samples and is potentially more appropriate for time series experiments such as human degenerative disease or bacterial pathogenesis progress where the condition of a patient/host changes over time. In addition, availability of our method for a much wider range of organisms is another advantage to GSEA that is just available for limited model organisms.

In contrast to other web servers [3, 5], our web server provides interactive visual navigation along the hierarchical structure of GO graphs at all levels of the graph. Furthermore, our web server provides dynamic visual reports (using AJAX technology) including pie charts (to visualize GO enrichment proportions) and bar charts (to visualize over-representation analysis), whereas other web servers present this information in text format or rely on visualization capacity provided by other websites including The European Bioinformatics Institute at http://www.ebi.ac.uk/.

The most significant analytical advantage provided by our web server is the ability to enrich and compare GO terms between multiple gene samples from multiple biological conditions. At present, other web servers [3, 5] can only compare one sample against a control sample. Comparative GO analysis is important as a means to identify underlying biological pathways involved in response to different biological conditions. This is essential if one wishes to identify candidate genes for perturbation experiments.

From a technical point of view, special caching, connection pooling and database query planning and optimization techniques were employed to make the webserver capable of accepting very large lists of genes such as the entire human transcriptome.

We demonstrated the efficiency of our proposed method in prokaryote and eukaryote case studies.

In the case of AD samples, the outlier test revealed upregulation of inflammation related function in AD brain such as granulocyte colony-stimulating factor receptor binding (governed by *CSF3*). *CSF* genes are pro-inflammatory cytokines which are expressed in brain and nervous system disorders and are involved in immunity and inflammation by regulating survival and proliferation. CSF proteins can pass through the blood–brain barrier and influence nervous system activities such as axonal regeneration [26, 27]. *CSF3* can transit between blood and brain and it shows a remarkable increase of its function in AD brain, so we hypothesise that *CSF3* might be tested as a blood based marker of AD in future studies. Another upregulated functional group was Interleukin-1 (IL-1), a pro-inflammatory cytokine that activates many inflammatory processes with important functions in brain neuroimmune responses. IL-1 has been evaluated as a target for therapeutic strategies using Interleukin-1 receptor antagonists in stroke and neural disorders [28–30]. It has also been reported that soluble interleukin-1 receptor increases in the cerebrospinal fluid of AD patients [31]. Interleukin-1 expression in brain can activate caspase-1 and apoptosis. The brain specific mechanisms of action of Interleukin-1 are not yet fully characterised, but may affect glia, endothelia, and neurons [28–30]. Whole transcriptome Gene Ontology based analysis in this study reinforces the hypothesis that inflammatory process are part of the neuropathology in AD. Overall, in comparison to GSEA analysis results that only highlighted already described secondary sensory effects, our method had the ability to introduce new mechanism and new target genes for AD.

Whole transriptome GO comparison of highly pathogenic *Salmonella enteritidis* compared to low pathogenic *S*. *enteritidis* highlighted the key roles of bacterial-type flagellum-dependent cell motility and chemotaxis in highly pathogenic Salmonella. Chemotaxis is biological process dependent on signal transduction and phosphorylation. We speculate that up regulating GO “Signal transduction by phosphorylation” may allow *Salmonella enteritidis* to more rapidly sense environmental changes and activate more genes through increased phosphorylation activity. It has been documented that chemotaxis is central for virulence and competitive fitness of *Ralstonia solanacearum* [32]. *Ralstonia solanacearum* has a remarkable capability for invading host plant roots from the soil to get amino acids and organic acids [32].

Motility has been identified as key virulence factor in bacteria as many bacteria use flagella to move and cause diseases in humans, animals and plants [33]. In line with our finding on the key roles of flagellum and motility in *S*. *enteritidis* pathogenicity, it has been demonstrated in *E*. *coli* that mobile genetic elements and flagellum motility are molecular mechanisms which contribute in increasing *E*. *coli* pathogenicity to generate a highly adapted pathogen capable of causing a range of diseases in the central nervous system, the gastrointestinal tract, the urinary tract, and blood [34]. Regarding the key roles of flagellum filaments and flagellum-dependent cell motility in bacterial pathogenicity, bacterial genes encoding filament components have been used for vaccine development and therapeutic interventions [35]. *Fli* genes in *S*. *typhimurium*, *Bacillus subtils* and *E*. *coli* are the major loci in flagellum biogenesis [36]. Flagellum and motility are also central for invasion of fish hosts by *Vibrio anguillarum* as disruption of the flagellum and loss of motility decreased virulence by 500-fold [37].

We have used our method with mRNA values, but it can also be used with protein values. Using protein abundance would yield even more accurate GO enrichment scores.

The new global transcriptomics, multi-sample GO enrichment methods presented in this report and implemented in the Comparative GO Web application [17] can significantly help to develop new hypotheses for further experiments. The method has the potential to improve bacterial regulatory mechanisms and eukaryotic functional genomics.

## Supporting information

S1 File Whole transcriptome expression levels (RPKM) of low and high pathogenic *Salmonella enteritidis*. (XLSX)

S2 File Whole transcriptome expression levels (RPKM) of normal and Alzheimer’s disease of human samples. (XLSX)

S3 File GSEA analysis result on Alzheimer’s disease of human samples. (XLSX)
